# Supplementary material for: A Global Screen for Assembly State Changes of the Mitotic Proteome by SEC-SWATH-MS
Source: Cell Syst. 2020 Feb 26;10(2):133–155.e6. doi: 10.1016/j.cels.2020.01.001 (PMC7042714; doi:10.1016/j.cels.2020.01.001)

P63208 | SKP1_HUMAN | SKP1 EMC19 OCP2 SKP1A TCEB1L
Monomer MW [kDa]: 18.658 Monomer expected elution fraction: 55

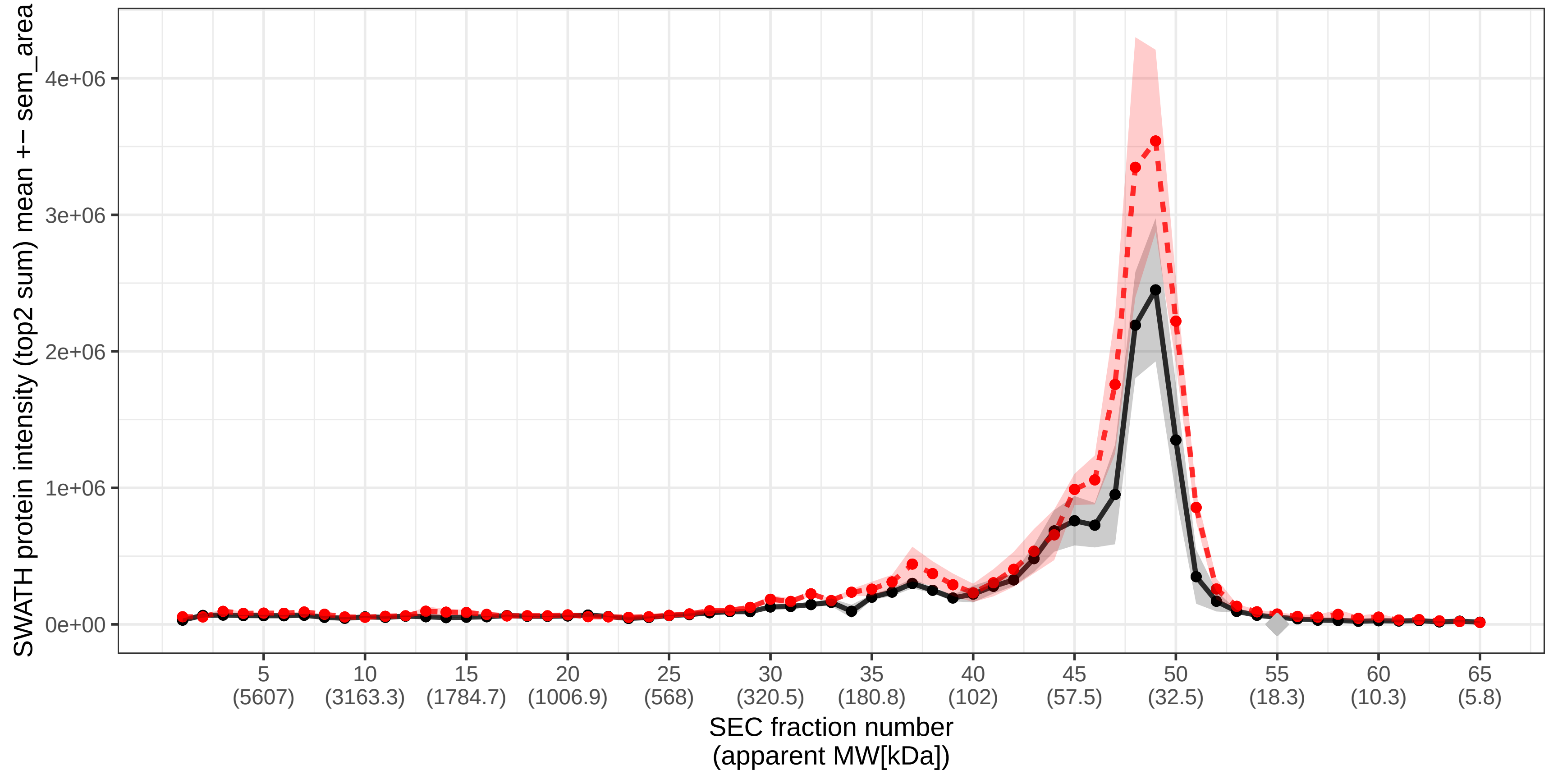

Supplement: Data S1. SEC-SWATH-MS Protein Chromatograms, Related to Figure 1 [file mmc6.zip › SECchrom_P63208_SKP1_HUMAN_SKP1_EMC19_OCP2_SKP1A_TCEB1L.pdf]
